# Characterization of remission levels in patients with ulcerative colitis treated with 5-aminosalicylates: results of the cross-sectional CARUC-ASA study by the Belgian IBD Research and Development Group

**DOI:** 10.1093/ecco-jcc/jjag100

**Published:** 2026-08-09

**Authors:** Catherine Reenaers, Guy Lambrecht, Lieven Pouillon, Filip Baert, Anneline Cremer, Arnaud Colard, Christophe Claessens, Thomas Billiet, Jeroen Geldof, Astrid De Zutter, Jean-François Rahier, Michael Somers, Dries Reynders, Laura Vansteenkiste, Ingrid Arijs, Edouard Louis, on behalf of the Belgian IBD Research and Development Group (BIRD), Bram Verstockt

**Affiliations:** Department of Gastroenterology, University Hospital of Liège, Liège University, Liège, Belgium; Department of Gastroenterology, AZ Oostende, Oostende, Belgium; Department of Gastroenterology, Imelda Bonheiden, Bonheiden, Belgium; Department of Gastroenterology, AZ Delta, Roeselare, Belgium; Department of Gastroenterology, HUB Erasme Bruxelles, Anderlecht, Belgium; Department of Gastroenterology, CHC MontLégia Liège, Belgium; Department of Gastroenterology, AZ Turnhout, Turnhout, Belgium; Department of Gastroenterology, AZ Groeninge, Groeninge, Belgium; Department of Gastroenterology, Gent university, Gent, Belgium; Department of Gastroenterology, Sint-Andriesziekenhuis, Tielt, Belgium; Department of Gastroenterology, CHU UCL Namur, Université catholique de Louvain, Yvoir, Belgium; Department of Gastroenterology, University Hospital Antwerpen, Antwerpen, Belgium; Department of Mathematics, Computer Science and Statistics, Gent University, Gent, Belgium; Belgian Inflammatory Bowel Disease Research and Development (BIRD); Belgian Inflammatory Bowel Disease Research and Development (BIRD); Department of Gastroenterology, University Hospital of Liège, Liège University, Liège, Belgium; Department of Gastroenterology and Hepatology, University Hospitals Leuven, KU Leuven, Leuven, Belgium; Department of Chronic Diseases and Metabolism, KU Leuven, Leuven, Belgium

**Keywords:** 5-ASA, ulcerative colitis, remission status, deep remission

## Abstract

**Background:**

5-Aminosalicylic acid (5-ASA) remains the first-line therapy for mild-to-moderate ulcerative colitis (UC). Modern treatment goals extend beyond symptom control to include steroid-free remission, endoscopic healing, and biomarker normalization. Real-world evidence on 5-ASA’s ability to achieve these targets is limited. This study assessed remission levels and associated factors in Belgian UC patients in clinical remission on 5-ASA.

**Methods:**

This cross-sectional, multicenter, phase 4 study included UC patients in clinical remission (PRO-2 ≤ 1, no rectal bleeding) treated with 5-ASA for ≥6 months. Patients receiving corticosteroids, immunomodulators, or advanced therapies were excluded. Outcomes were complete clinical remission (PRO-2 = 0, no urgency), endoscopic remission (MES ≤ 1, UCEIS ≤ 1), complete endoscopic remission (MES = 0), histological remission (Geboes 0-1), biological remission (fecal calprotectin <150 µg/g), and deep remission (clinical, endoscopic, and histological). Adherence was assessed using MARS-5.

**Results:**

In total, 198 patients were enrolled (median age 50 years; 41% female). Treatment was oral 5-ASA only (*n* = 134, 68%), rectal only (*n* = 19, 9%), or combination (*n* = 45, 23%). Biological remission was observed in 78%, endoscopic remission in 87%, complete endoscopic remission in 70%, and histological remission in 80%. Deep remission occurred in 24%, limited by persistent urgency (63%). Adherence was high (median MARS-5: 24). Oral 5-ASA only was significantly associated with endoscopic remission (*P* < .001).

**Conclusion:**

In this real-world Belgian cohort, patients in clinical remission on 5-ASA achieved high rates of objective remission, while deep remission was low because of persistent urgency. These findings confirm 5-ASA’s continued relevance in mild-to-moderate UC and highlight the importance of addressing residual symptoms despite mucosal healing.

## 1. Introduction

5-Aminosalicylic acid (5-ASA) compounds remain the cornerstone of treatment for patients with mild-to-moderate ulcerative colitis (UC) despite the advent of biological agents and small molecules.^1^ For many patients, 5-ASA is effective for both induction and maintenance of remission,^2^ with up to 50% achieving long-term disease control on 5-ASA monotherapy throughout their disease course.^3^^,^^4^ Optimal use of 5-ASA, through appropriate formulation selection (oral and/or rectal) and adequate dosing, can maximize efficacy and potentially delay escalation to advanced therapies, which are associated with significantly higher costs and eventually increased risk of adverse effects.^5^^,^^6^

The STRIDE II consensus redefined treatment targets in UC, advocating for targets beyond symptom control, including endoscopic remission, normalization of biomarkers, and restoration of health-related quality of life.^7^ Although histological remission is not yet universally endorsed as a formal treatment target, growing evidence suggests that it is associated with a reduced risk of relapse, hospitalization, and colectomy.^8^

While these therapeutic endpoints are now routinely assessed in clinical trials of advanced therapies, there is limited real-world evidence on the extent to which 5-ASA can achieve such comprehensive targets, particularly when considering the full spectrum from clinical remission to deep remission. Few studies have systematically evaluated histological, endoscopic, biological, and complete clinical remission simultaneously in patients on 5-ASA monotherapy.^9^

This study aimed to address this gap by providing real-world data from Belgian patients with UC in clinical remission on 5-ASA. The primary objective was to quantify the proportion of patients achieving complete clinical, biological, endoscopic, and histological remission during routine follow-up. Secondary objectives included identifying factors associated with failure to achieve deep remission, assessing treatment adherence and its impact on outcomes, and exploring the relationship between 5-ASA regimen (dose and formulation) and remission. Additionally, we assessed health-related quality of life and physician-reported reasons for persistent disease activity in patients without endoscopic remission.

## 2. Patients and methods

### 2.1. Study population

Patients with a confirmed diagnosis of UC, currently receiving 5-ASA monotherapy (either orally, rectally, or both), seen during a routine visit, were eligible for inclusion. Inclusion criteria were: age ≥18 years, in clinical remission, defined as a two-item Patient Reported Outcome (PRO-2) score ≤ 1 (stool frequency ≤ 1 and rectal bleeding score = 0 over the preceding 3 days). Patients were required to be treated with 5-ASA for at least 6 months prior to inclusion, while the dose had to remain unchanged for at least 2 weeks before study entry. Self-medication, including corticosteroids, was assessed during the study visit through a structured review of the patient’s current treatment.

Exclusion criteria included: history of total colectomy (with or without an ileal pouch anal anastomosis), pregnancy, inflammatory bowel disease (IBD) type unclassified, or use of corticosteroids, immunomodulators, biologics, Janus kinase (JAK) inhibitors, sphingosine-1-phosphate receptor (S1PR) modulators, or investigational drugs within the previous 3 months as well as hospitalization or urgent outpatient visit in the last 3 months.

### 2.2. Study design

This was a phase 4, national, multicenter, cross-sectional, interventional study conducted in Belgian IBD Research and Development (BIRD) centers (ClinicalTrials.gov: NCT05992142). Fourteen academic and nonacademic sites participated, with enrollment planned for 200 patients over an 18-month period.

Screening and inclusion occurred during a single study visit. Demographic data, medical history, UC characteristics, and current and previous treatments were collected.

### 2.3. Assessments

PRO-2 scores were calculated as the mean of stool frequency and rectal bleeding over the preceding 3 days. Bowel urgency during the prior 24 h was assessed using the Ulcerative Colitis Numeric Rating Scale (UNRS) ranging from 0 (no urgency) to 10 (worst possible urgency).^10^

Fecal calprotectin (FC) and flexible rectosigmoidoscopy were performed at the visit, or within 2 weeks, provided no UC-related medical intervention occurred in the interim. FC was measured in local laboratories according to standard protocols. In active disease, two biopsies were taken from the most inflamed area; if endoscopy appeared normal, samples were taken from the rectum. The Mayo Endoscopic Subscore (MES) and Ulcerative Colitis Endoscopic Index of Severity (UCEIS) were assessed locally. Central histological review was performed at the University Hospital of Liège by a blinded expert pathologist (JS), using the Geboes score.

Quality of life was assessed using the Short Health Scale (SHS), a validated visual analog scale evaluating disease burden, functional limitations in daily life, disease-related worry, and general well-being.^11^

Adherence was assessed by the MARS-5 (five-item Medication Adherence Report Scale) questionnaire, a scale including five statements describing nonadherent behaviors.^12^ For patients without complete endoscopic remission, physicians were allowed to modify their therapeutic strategy. An exploratory questionnaire on therapeutic strategy and reasons for not modifying treatment was completed by the physician.

### 2.4. Definitions

Clinical remission was defined as PRO-2 score ≤ 1 (stool frequency ≤ 1 and rectal bleeding score = 0 over the preceding 3 days). Complete clinical remission was defined as a PRO-2 score of 0 and the absence of bowel urgency (0 on UNRS). Partial clinical remission was defined as the absence of complete clinical remission. Endoscopic remission was defined as MES ≤ 1 and UCEIS ≤ 1 and complete endoscopic remission as MES = 0 and/or UCEIS = 0. Histological remission was defined as absence of acute inflammatory activity on biopsy, corresponding to a Geboes score of 0 (inactive disease) or 1 (chronic inflammation). Biological remission was defined as FC < 150 µg/g. Deep remission was defined as the combination of complete clinical, complete endoscopic, and histological remission.

Patients were considered adherent if they scored ≥21 on the total MARS scale, or ≥4 on each individual item. Regarding oral 5-ASA dosage, <2 g/day was summarized as “low dose,” 2-3 g/day as “standard dose,” and 4 g/day as “high dose.” For rectal 5-ASA dosage, <1 g/day was categorized as “low dose,” 1 g/day as “standard dose,” and >1 g/day as “high dose.”

### 2.5. Statistical analysis

No formal statistical sample size calculation was performed because the study was designed as a single-arm study without a hypothesis testing framework or a planned comparison between groups. The target enrollment of 200 patients over an 18-month period was determined pragmatically to obtain a sufficiently large and representative sample of the study population and to ensure adequate descriptive analysis of the outcomes. As the primary aim of the trial was descriptive, no formal sample size calculation was performed. Remission rates were summarized using uni- and bivariate analyses. Logistic regression was used to assess associations between remission and 5-ASA formulation, adherence, corticosteroid use, demographics (sex and smoking status), and disease extent; linear regression was applied for quality-of-life (QoL) outcomes. QoL was compared between partial and complete clinical remission using the Wilcoxon test. No adjustment was made for multiple comparisons.

## 3. Results

### 3.1. Patient characteristics

Between January 2023 and February 2024, 200 patients were screened; two were excluded due to protocol violations: one for initiating 5-ASA therapy less than 6 months prior to inclusion and corticosteroid use within the last 3 months, and one for corticosteroid use within the last 3 months prior to inclusion. The final analysis included 198 patients. Detailed demographic and therapeutic data are summarized in Table 1. All patients were naive from advanced treatment. No patients had a history of acute severe UC.

**Table 1.** Patient characteristics and 5-aminosalicylic acid (5-ASA) treatment modalities.

Patient characteristics (*n* = 198)	*n* (%); median (IQR)
**Male**	116 (59)
**Age, years**	50 (38, 63)
**Smoking habit**	
** Smoker (>1 cigarette/day)**	12 (6.1)
** Former smoker**	73 (37)
** Non-smoker**	113 (57)
**Disease duration**	7 (3, 15)
**Montreal classification**	
** E1**	73 (37)
** E2**	87 (44)
** E3**	36 (18)
** Pancolitis**	23 (12)
** Unknown**	2 (1)
**Extra-intestinal manifestations**	
** Ankylosing spondylitis**	1 (0.5)
** Sclerosing cholangitis**	2 (1.0)
**Corticoid use 3-12 months before inclusion**	17 (8.6)
**5-ASA regimen**	
** Oral**	134 (68)
** Topic**	19 (9.6)
** Oral + topic**	45 (23)
**Dosage oral 5-ASA monotherapy (*n* = 134)**	
** Low (<2 g/day)**	27 (20)
** Normal (2-3 g/day)**	84 (63)
** High (>3 g/day)**	23 (17)
**Dosage rectal 5-ASA**	
** Low (<1 g/day)**	11 (58)
** Normal (1 g/day)**	8 (43)
** High (>1 g/day)**	0 (0)
**Duration of current 5-ASA treatment (years)**	2.00 (1.00-4.00)

### 3.2. Remission outcomes

Complete clinical remission was observed in 37% of the patients (*n* = 74). Among the 124 without complete clinical remission, 31% had PRO-2 > 0, and 85% reported bowel urgency (median bowel urgency score: 3 [IQR 1-4]).

Biochemical remission was present in 78% of the patients (152/194 patients) with data missing for four patients.

**Figure 1.**
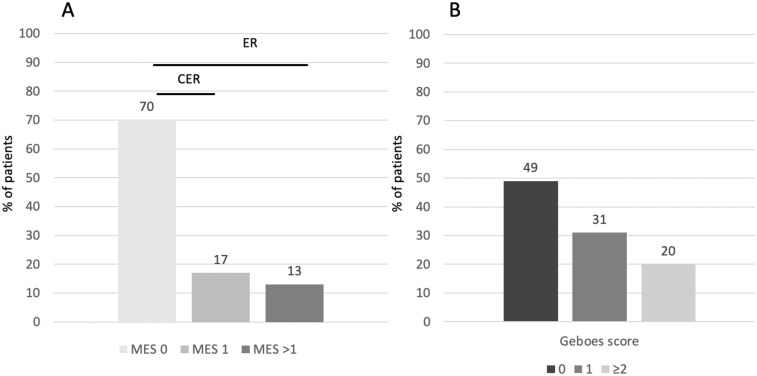
Endoscopic and histological remission. (A) MES in the whole population. In total, 87% of patients achieved endoscopic remission, defined by an MES ≤ 1 and 70% complete endoscopic remission, defined by an MES = 0 and UCEIS = 0. MES, Mayo Endoscopic Subscore; UCEIS, Ulcerative Colitis Endoscopic Undex of Severity; ER, endoscopic remission; CER, complete endoscopic remission. (B) Histological Geboes score in the whole population. In total, 49% of patients had no signs of activity (Geboes = 0), 31% had chronic signs of inflammation (Geboes = 1), and 20% had acute signs of activity (Geboes ≥ 1). In total, 80% of patients achieved histological remission.

Endoscopic remission was observed in 87% of the patients (180/198) with complete endoscopic remission in 70% (138/198). Moderate (MES = 2) and severe (MES = 3) endoscopic activity were reported in 8% and 1%, respectively (Figure 1A).

Histological remission was present in 80% of the patients (156/196) including 49% with a Geboes score of 0, and 31% with a score of 1. Acute histological inflammation (Geboes score ≥ 2) was found in 20%, distributed as follows: grade 2 in five patients (2.5%), grade 3 in 25 (12.5%), grade 4 in five (2.5%), and grade 5 in five (2.5%). Biopsies were missing for two patients (Figure 1B).

**Figure 2.**
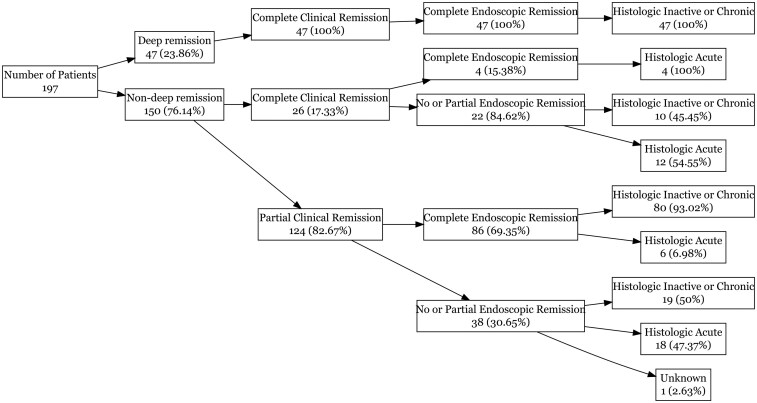
Flowchart illustrating the reasons for not achieving deep remission. A total of 197 patients were evaluated. Among the 150 patients with absence of deep remission, the reason was the persistence of clinical symptoms in 83% of cases. In the 26 patients with complete clinical remission the main reason for not achieving deep remission was the absence of complete endoscopic remission in 85% of cases. Deep remission was not assessable for one patient (histological results not available).

Deep remission was seen in 24% of patients (*n* = 47). The main limiting factor for absence of deep remission was absence of complete clinical remission in 124 patients (63%), predominantly due to bowel urgency. A detailed flowchart of reasons for not achieving deep remission is provided in Figure 2.

### 3.3. Associations between the different levels of remission

**Figure 3.**
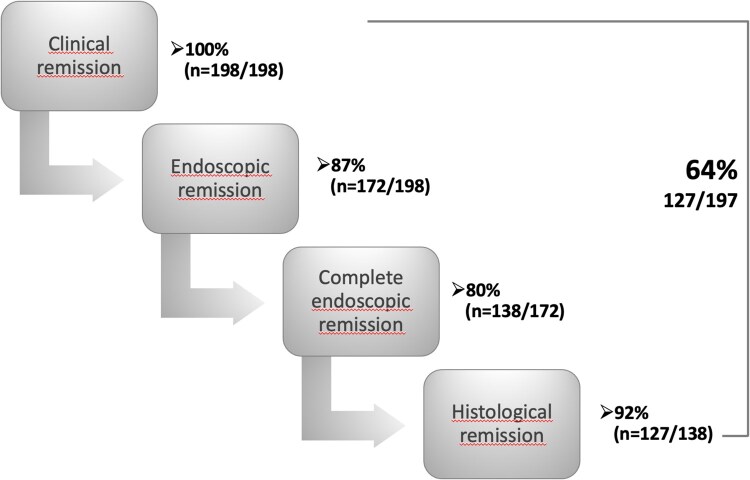
Proportions of patients who achieved endoscopic remission, complete endoscopic remission, and histological remission. The figure illustrates the stepwise decrease in remission rates with increasingly stringent definitions. The combination of the levels of remission was achieved in 127 patients (64%).

Of the 172 patients in endoscopic remission, 80% (*n* = 138) also had complete endoscopic remission and 92% of these (127/138) had histological remission. In the total population, 64% (*n* = 127/198) exhibited both complete endoscopic and histological remission (Figure 3). Vice versa, among the 156 patients with histological remission, 151 (97%) also had endoscopic remission, and 127 (81%) had complete endoscopic remission (Figure 3).

**Figure 4.**
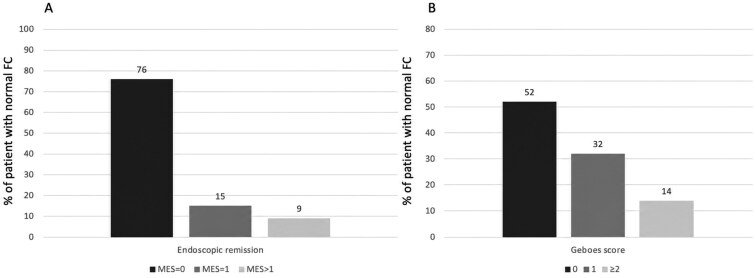
Proportion of patients with normal fecal calprotectin (FC) stratified by remission status. (A) Distribution of endoscopic remission status among patients with normal FC levels. (B) Distribution of histological remission status based on Geboes score among patients with normal FC levels.

Among patients in biological remission, 91% (*n* = 138/152) had endoscopic remission, and 76% (*n* = 115/152) had complete endoscopic remission (Figure 4A). Similarly, 85% of patients in biological remission (*n* = 128) had histological remission (Figure 4B).

### 3.4. Adherence and quality of life

Adherence, as assessed using the MARS-5 questionnaire, demonstrated a median score of 24 (IQR: 23-25), with significantly higher adherence for oral therapy only compared to other formulations (*P* < .001).

Regarding QoL, median SHS scores for the following four domains were as follows: “symptoms”: 10 (IQR: 0-22); “impact on decision-making in patients’ lives”: 1 (IQR: 0-12); “worry about the disease”: 10 (IQR: 0-32); and “general well-being”: 11 (IQR: 0-30). Across all four domains, scores were higher in patients with complete versus partial clinical remission (*P* = .002, *P* = .003, *P* = .003, and *P* = .012, respectively). Similar differences—in terms of both magnitude and statistical significance—were observed for endoscopic, complete endoscopic, histologic, and deep remission. For biological remission, the observed differences were more moderate and most were not significant.

### 3.5. Factors associated with the different levels of remission

Regarding factors associated with endoscopic outcomes, both oral 5-ASA therapy compared to rectal only and oral + rectal (*P* = .05; OR = 2.90; 95% CI: 1.14-7.31) and disease extent classified as Montreal E2, compared to E3 (*P* = .01; OR = 4.25; 95% CI: 1.55-13.6), were significantly associated with endoscopic remission. No variables were found to be associated with complete endoscopic remission or with histological remission. Higher adherence was associated with higher rates of complete endoscopic (*P* = .05; OR = 1.12; 95% CI: 1.00-1.26) and histological remission (*P* = .06; OR = 1.12; 95% CI: 0.99-1.27). Full details are presented in Table 2.

**Table 2.** Factors associated with the different levels of remission.

Variable	*P*	OR	95% CI
**Endoscopic remission**			
**Sex**	0.26	0.62	0.26-1.43
**Smoking status**	0.15		
** Active**		–	–
** Non-smokers**		0.00	
** Former smokers**		0.00	
**Montreal classification (E2 vs E3)**	0.01		
** 1**		4.24	1.50-13.60
** 2**		0.58	0.16-2.38
** 3**		2.72	0.69-18.20
**Disease duration**	0.68	0.99	0.96-1.04
**5-ASA administration form**	0.05		
** Combined**		–	–
** Oral**		2.90	1.14-7.31
** Rectal**		1.07	0.30-4.39
**Oral 5-ASA dosage (low vs high)**	0.30	0.86	0.15-4.37
**Exposure to CS 3-12 months before inclusion**	0.86	1.15	0.30-7.56
**MARS score**	0.19	1.10	0.95-1.25
**Complete endoscopic remission**			
**Sex**	0.58	0.84	0.45-1.57
**Smoking status**	0.58		
** Active**		–	–
** Non-smokers**		0.47	0.07-1.93
** Former smokers**		0.45	0.07-1.91
**Montreal classification (E2 vs E3)**	0.96		
** 1**		1.07	0.54-2.12
** 2**		0.97	0.28-3.89
** 3**		0.81	0.30-2.26
**Disease duration**	0.82	1.00	0.97-1.03
**5-ASA administration form**	0.84		
** Combined**		–	–
** Oral**		1.18	0.56-2.40
** Rectal**		1.40	0.44-4.99
**Oral 5-ASA dosage (low vs high)**	0.45	1.29	0.4-4.15
**Exposure to CS 3-12 months before inclusion**	0.32	0.56	0.22-1.71
**MARS score**	0.05	1.12	1.00-1.26
**Histological remission**			
**Sex**	0.56	1.23	0.61-2.59
**Smoking status**	0.88		
** Active**		–	–
** Non-smokers**		0.76	0.11-3.21
** Former smokers**		0.68	0.10-2.96
**Montreal classification (E2 vs E3)**	0.96		
** 1**		0.88	0.39-1.95
** 2**		0.49	0.13-2.01
** 3**		0.58	0.19-1.86
**Disease duration**	0.80	1.00	0.96-1.03
**5-ASA administration form**	0.10		
** Combined**		–	–
** Oral**		2.03	0.93-4.37
** Rectal**		3.84	0.92-26.3
**Oral 5-ASA dosage (low vs high)**	0.57	1.94	0.48-8.65
**Exposure to CS 3-12 months before inclusion**	0.13	0.43	0.13-1.32
**MARS score**	0.06	1.12	0.99-1.27

Abbreviations: OR, odds ratio; CI, confidence interval; 5-ASA, 5-aminosalicylic acid; CS, corticosteroids; MARS 5, five-item Medication Adherence Report Scale.

### 3.6. Physician and patient perspectives for absence of deep remission

Among the 60 patients without complete endoscopic remission, physician questionnaires were completed for 55 patients to assess whether a treatment change would be considered based on the endoscopic findings. Therapy was modified in 24 cases (dose increase and/or formulation change), and 5-ASA was stopped in favor of another treatment in two cases.

In addition, 27 questionnaires were completed to explore the reasons for not modifying treatment. The main reasons for not modifying treatment were as follows: the physician judged that a step-up in therapy was not warranted in the current context (*n* = 20), poor patient adherence (*n* = 6), and patient refusal to change treatment (*n* = 1).

## 4. Discussion

This multicenter, cross-sectional study provides a comprehensive overview of remission status in a Belgian cohort of patients with UC receiving 5-ASA monotherapy. We reported high rates of endoscopic (87%), histological (80%), and biological (78%) remission with 64% of patients in the total study population achieving both complete endoscopic and histological remission. These numbers are significantly higher than those documented in several maintenance studies across various therapeutic classes. For instance, in the long-term extension of the UNIFI study looking at the long-term efficacy of ustekinumab in UC after 4 years, 55% of patients randomized to ustekinumab at maintenance baseline were in symptomatic remission at week 200 and 67.2% among biologic-naive patients. A total of 67% of patients had endoscopic improvement at week 200 defined in the trial as MES < 2. Similarly, histological remission, observed in 80% of our cohort, exceeds the rates typically reported with biologics, which generally range from 20% to 40% in phase 3 trials, depending on the definition used and the patient population studied.^6^^,^^13^

This high efficacy seen here probably reflects differences in study populations. Unlike trials of biologics or small molecules, our cohort included bionaive, mild-to-moderate UC patients with less complicated disease courses. Although 9% had received corticosteroids within the prior 3-12 months before inclusion, all had successfully discontinued them, and recent corticosteroid use was not associated with poorer remission outcomes. This suggests that in appropriately selected patients, 5-ASA remains highly effective and consistent with STRIDE-II targets of clinical and endoscopic remission, with histological remission as a valuable adjunctive goal. In this regard, our cohort demonstrates substantial alignment with STRIDE-II targets, with over two-thirds of patients ­meeting both clinical and endoscopic remission criteria and 64% achieving combined endoscopic and ­histological remission.

The efficacy of 5-ASA treatment relies not only on the appropriate formulation and dosing, but also on sustained patient adherence, usually defined as taking at least 80% of the prescribed medication. In our study, a relatively high proportion of patients received rectal therapy as maintenance treatment (1 g/day, 43%). This probably reflects the distribution of disease extent in our cohort, which included a substantial proportion of patients with distal UC (proctitis or left-sided disease). This practise is consistent with current ECCO guidelines, which recommend topical 5-ASA as maintenance therapy for distal disease, either as monotherapy or in combination with oral 5-ASA.^2^

Ensuring long-term adherence remains a well-recognized challenge in chronic conditions, with real-world adherence rates often reported between 50% and 70%.^14–18^ In our study, adherence was notably high, with a median MARS-5 score of 24. The predominance of oral 5-ASA monotherapy in this cohort may have contributed, as adherence is generally higher for oral than rectal formulations. Prantera et al. reported similar data with higher adherence to oral 5-ASA compared to rectal therapy (97% vs 87.5%).^17^

Despite high rates of objective remission in our cohort, only 24% of patients met the criteria for deep remission, defined as the combination of complete endoscopic and histological healing along with the absence of clinical symptoms. Among the 198 patients assessed, 124 were excluded from the deep remission group due to persistence of clinical symptoms (including bowel urgency in 85%), although 80 of them exhibited both endoscopic and histological remission. Including these patients would have raised the deep remission rate to 64% (127/196), further highlighting the high objective remission rates observed. Persistent irritable bowel syndrome (IBS)-like symptoms are well documented in Crohn’s disease^17^ and are increasingly recognized in UC.^19^ In the Norwegian IBSEN cohort, 33% of UC patients in deep remission continued to report IBS-like symptoms 20 years after diagnosis.^20^ A meta-analysis by Halpin et al. found a pooled IBS prevalence of 39% among patients with IBD, with higher rates in Crohn’s disease than in UC (46% vs 36%).^21^ Beside IBS-like symptoms, UC patients can report bowel urgency in up to 32-35% of the cases.^22^^,^^23^ The high prevalence of bowel urgency observed in our cohort probably reflects the structural and functional alterations of the colon and rectum associated with UC. Mucosal inflammation may lead to reduced rectal compliance, altered rectal sensation, and increased hypersensitivity, which contribute to urgency. These mechanisms differ from IBS-like symptoms, which are mainly related to disorders of gut–brain interaction rather than inflammatory structural changes.^10^ As in this study, bowel urgencies may be observed even in patients with endoscopic or histological remission and it has a substantial negative impact on health-related QoL. Identifying these patients is essential to avoid unnecessary therapeutic escalation and to promote more appropriate, treat-to-targeted strategies.

Another important observation was the relatively low rate of treatment modification in patients without complete endoscopic remission. Only 50% of patients with MES ≥ 1 underwent therapeutic modification, often limited to optimizing 5-ASA dosage. In clinical practise, endoscopic remission is often defined as MES of 0 or 1,^24^ which may have influenced clinicians’ decisions to refrain from escalating treatment in such cases. Conflicting data exist regarding the prognostic value of MES 0 versus MES 1 in UC. The meta-analysis from Yoon et al. (>2000 patients) reported a significantly lower relapse risk with MES 0 (hazard ratio [HR] ∼ 0.48, 95% CI 0.37-0.62).^25^ More recently, in a prospective cohort, the 6-month relapse rate was significantly lower in patients with MES 0 (9.4%) compared to those with MES 1 (36.6%) (OR 6.27; *P* < .001).^26^ In two recent studies, Kim et al.^27^ and Laterza et al.^28^ found only non-significant trends (HR 0.72 and HR 0.89, respectively) in favor of MES 0 over MES 1 after a respective follow-up of 2 and 3 years. Nevertheless, in real-world settings, particularly among patients with MES 0 or 1 under 5-ASA, clinicians may weigh the uncertain benefit of escalation in asymptomatic patients against potential risks and costs. Reimbursement issues might also refrain physicians from upscaling in this situation. Prospective follow-up of this subgroup is warranted to better characterize long-term clinical outcomes.

This study has several limitations. The inclusion of patients was not based on consecutive patient inclusions. Its cross-sectional design does not allow for assessment of remission durability or the ability to predict future relapse. Endoscopic assessments were performed locally, without central reading, and adherence was self-reported, which may overestimate true adherence. Studies have shown that adherence tends to be lower when measured using objective methods, such as pharmacy refill data or electronic monitoring.^29^ Despite this limitation, the high levels of clinical, endoscopic, and histological remission observed in our cohort support the likelihood of global good adherence. Finally, our cohort included only patients already stable on 5-ASA monotherapy, enriching for responders and limiting extrapolation to the general UC population.

In conclusion, 5-ASA monotherapy can achieve high rates of endoscopic and histological remission in a real-world setting, particularly in patients with limited disease extent and high adherence. These outcomes are consistent with STRIDE-II therapeutic targets and underscore the continued relevance of 5-ASA as a cornerstone treatment for mild to moderate UC. Future studies should investigate whether early identification of subclinical inflammation or incomplete symptom resolution should prompt therapeutic adaptation to improve long-term outcomes in patients on 5-ASA.

## Data Availability

The data that support the findings of this study are available as individual anonymized data upon request to the corresponding author.
